# Poliovirus receptor–based chimeric antigen receptor T cells combined with NK-92 cells exert potent activity against glioblastoma

**DOI:** 10.1093/jnci/djad226

**Published:** 2023-11-07

**Authors:** Changqing Pan, You Zhai, Chen Wang, Zhiyi Liao, Di Wang, Mingchen Yu, Fan Wu, Yiyun Yin, Zhongfang Shi, Guanzhang Li, Tao Jiang, Wei Zhang

**Affiliations:** Department of Neurosurgery, Beijing Tiantan Hospital, Capital Medical University, Beijing, PR China; Department of Neurosurgery, Beijing Tiantan Hospital, Capital Medical University, Beijing, PR China; Department of Molecular Neuropathology, Beijing Neurosurgical Institute, Capital Medical University, Beijing, PR China; Department of Neurosurgery, Beijing Tiantan Hospital, Capital Medical University, Beijing, PR China; Department of Molecular Neuropathology, Beijing Neurosurgical Institute, Capital Medical University, Beijing, PR China; Department of Neurosurgery, Beijing Tiantan Hospital, Capital Medical University, Beijing, PR China; Department of Molecular Neuropathology, Beijing Neurosurgical Institute, Capital Medical University, Beijing, PR China; Department of Molecular Neuropathology, Beijing Neurosurgical Institute, Capital Medical University, Beijing, PR China; Department of Molecular Neuropathology, Beijing Neurosurgical Institute, Capital Medical University, Beijing, PR China; Department of Pathophysiology, Beijing Neurosurgical Institute, Capital Medical University, Beijing, PR China; Department of Neurosurgery, Beijing Tiantan Hospital, Capital Medical University, Beijing, PR China; Department of Molecular Neuropathology, Beijing Neurosurgical Institute, Capital Medical University, Beijing, PR China; Chinese Glioma Genome Atlas Network and Asian Glioma Genome Atlas Network, Beijing, PR China; Department of Neurosurgery, Beijing Tiantan Hospital, Capital Medical University, Beijing, PR China; Department of Molecular Neuropathology, Beijing Neurosurgical Institute, Capital Medical University, Beijing, PR China; Chinese Glioma Genome Atlas Network and Asian Glioma Genome Atlas Network, Beijing, PR China; China National Clinical Research Center for Neurological Diseases, Beijing, PR China; Center of Brain Tumor, Beijing Institute for Brain Disorders, Beijing, PR China; Research Unit of Accurate Diagnosis, Treatment, and Translational Medicine of Brain Tumors, Chinese Academy of Medical Sciences, Beijing, PR China; Department of Neurosurgery, Beijing Tiantan Hospital, Capital Medical University, Beijing, PR China; Department of Molecular Neuropathology, Beijing Neurosurgical Institute, Capital Medical University, Beijing, PR China; Chinese Glioma Genome Atlas Network and Asian Glioma Genome Atlas Network, Beijing, PR China; China National Clinical Research Center for Neurological Diseases, Beijing, PR China; Center of Brain Tumor, Beijing Institute for Brain Disorders, Beijing, PR China

## Abstract

**Background:**

Poliovirus receptor interacts with 3 receptors: T-cell immunoglobulin immunoreceptor tyrosine-based inhibitory motif, CD96, and DNAX accessory molecule 1, which are predominantly expressed on T cells and natural killer (NK) cells. Many solid tumors, including IDH wild-type glioblastoma, have been reported to overexpress poliovirus receptor, and this overexpression is associated with poor prognosis. However, there are no preclinical or clinical trials investigating the use of cell-based immunotherapies targeting poliovirus receptor in IDH wild-type glioblastoma.

**Methods:**

We analyzed poliovirus receptor expression in transcriptome sequencing databases and specimens from IDH wild-type glioblastoma patients. We developed poliovirus receptor targeting chimeric antigen receptor T cells using lentivirus. The antitumor activity of chimeric antigen receptor T cells was demonstrated in patient-derived glioma stem cells, intracranial and subcutaneous mouse xenograft models.

**Results:**

We verified poliovirus receptor expression in primary glioma stem cells, surgical specimens from IDH wild-type glioblastoma patients, and organoids. Accordingly, we developed poliovirus receptor–based second-generation chimeric antigen receptor T cells. The antitumor activity of chimeric antigen receptor T cells was demonstrated in glioma stem cells and xenograft models. Tumor recurrence occurred in intracranial xenograft models because of antigen loss. The combinational therapy of tyrosine-based inhibitory motif extracellular domain–based chimeric antigen receptor T cells and NK-92 cells markedly suppressed tumor recurrence and prolonged survival.

**Conclusions:**

Poliovirus receptor–based chimeric antigen receptor T cells were capable of killing glioma stem cells and suppressing tumor recurrence when combined with NK-92 cells.

IDH wild-type glioblastoma is the most common malignant tumor in the adult central nervous system, and its generally poor prognosis calls for novel therapies. Poliovirus receptor, a type I transmembrane glycoprotein, was originally identified because of its ability to permit poliovirus attachment (1,2). The receptor has emerged as a factor contributing to tumorigenesis and immunomodulation in multiple contexts (3). Poliovirus receptor is expressed at very low levels in normal brain tissue (4,5) and overexpressed in glioblastoma (5,6). Overexpression of poliovirus receptor has also been associated with poor prognosis in other solid tumors, including melanoma (7), colorectal carcinoma (8), and breast cancer (9).

Poliovirus receptor interacts with several poliovirus receptor–like proteins, including T-cell immunoglobulin immunoreceptor tyrosine-based inhibitory motif, CD96, DNAX accessory molecule 1, and CD112R (10). Within this signaling network, the interaction between poliovirus receptor and tyrosine-based inhibitory motif plays a particularly important role in immune inhibition. This interaction has been shown to inhibit T-cell activity indirectly through the alteration of dendritic cell activity (11). In addition, chimeric antigen receptor T-cell dysfunction associated with tyrosine-based inhibitory motif expression contributed to poor responses in patients with relapsed or refractory non-Hodgkin lymphoma (12). The poliovirus receptor and tyrosine-based inhibitory motif interaction can also directly inhibit the cytotoxicity of natural killer (NK) cells, and high tyrosine-based inhibitory motif expression is associated with exhaustion of tumor-infiltrating NK cells (13,14).

Antagonists of poliovirus receptor–like proteins have been proposed in several therapeutic strategies (10,15). Intratumor delivery of a recombinant nonpathogenic polio-rhinovirus chimera in patients with recurrent grade 4 malignant glioma resulted in an increased survival rate (16). Another study evaluated the efficacy of NK cells expressing a DNAX accessory molecule 1–based chimeric receptor targeting poliovirus receptor and CD112 in neuroblastoma cells (17). However, there has been no preclinical or clinical trials investigating the use of cell-based immunotherapies targeting poliovirus receptor in IDH wild-type glioblastoma.

Ligand- or receptor-based chimeric antigen receptors have shown encouraging results in the treatment of multiple forms of cancer (18,19). Here, we developed several poliovirus receptor targeting chimeric antigen receptor T cells based on classical single-chain fragment variables (scFv) and natural receptors of poliovirus receptor. Robust in vitro and in vivo cytotoxicity exerted by these chimeric antigen receptor T cells against glioma stem cells was demonstrated. We also found that poliovirus receptor targeting chimeric antigen receptor T cells could slow tumor growth in subcutaneous models of colorectal carcinoma and melanoma. Tumor recurrence occurred because of poliovirus receptor loss, which sensitizes tumor cells to be killed by NK-92 cells whose functions can be inhibited by poliovirus receptor–tyrosine-based inhibitory motif interaction. As expected, a combinational therapy including tyrosine-based inhibitory motif extracellular domain-based chimeric antigen receptor T cells and NK-92 cells markedly prolonged survival. These findings demonstrate that a combinatorial strategy using tyrosine-based inhibitory motif extracellular domain-based chimeric antigen receptor T cells and NK-92 cells is a potential immunotherapy for the treatment of IDH wild-type glioblastoma.

## Methods

For more details, please refer to the Supplementary Methods (available online).

### Patients

Archived tumor and normal brain tissues were obtained from Beijing Tiantan Hospital, Capital Medical University. This study complied with all relevant ethical regulations approved by the institutional review board of Beijing Tiantan Hospital, and informed consent was obtained from each participant (IRB, ID: KY 2020-093-04). The study was conducted in accordance with European Good Clinical Practice requirements as stated in the Declaration of Helsinki.

### Cytotoxicity assays

Glioma stem cells expressing fluorescent proteins were co-cultured with chimeric antigen receptor T cells at an Effector to Tumor ratio of 1:1 for 12, 24, and 48 hours or at an Effector to Tumor ratio of 1:8, 1:4, 1:2, and 1:1 for 24 hours. At each time point, the cells were collected, washed, and analyzed by flow cytometry. An Fluorescein Isothiocyanate or Phycoerythrin channel was used to distinguish chimeric antigen receptor cells from tumor. The ratio of chimeric antigen receptor T cells to tumor cells was calculated to determine the killing capacity of the chimeric antigen receptor T cells.

### In vivo tumor modeling

All mice were housed in specific pathogen-free conditions at a barrier facility at Beijing Tiantan Hospital. All animal handling, surveillance, and experimentation were performed in accordance with guidelines and approval from the Laboratory Animal Care facility of Beijing Tiantan Hospital (IRB, ID: 201904005). To generate an orthotopic xenograft model, BNI-19-1-S cells expressing luciferase were dissociated to single cells using Accutase (Millipore) and were injected into the frontal lobes of 5-week-old Non Obese Diabetes mice. Cg-Prkdc^scid^IL2rg^tm1Vst^/Vst mice (Vitalstar Biotechnology Co, Ltd). Details are described in the Supplementary Methods (available online).

### Statistical analysis

The statistical significance of differences between the 2 groups was determined with a *t* test. Kaplan–Meier survival curves were developed for survival analysis, and the log-rank test was used to test the statistical significance. Other statistical analyses included 1-way analysis of variance and 2-way repeated-measures analysis of variance tests. The results and the statistical analysis were performed using Prism (version 8.0) and R (version 4.0.0, http://www.r-project.org). All statistical tests were 2-sided, and a *P* value less than .05 was considered statistically significant.

## Results

### Poliovirus receptor is a potential target for IDH wild-type glioblastoma immunotherapy

**Figure 1.**
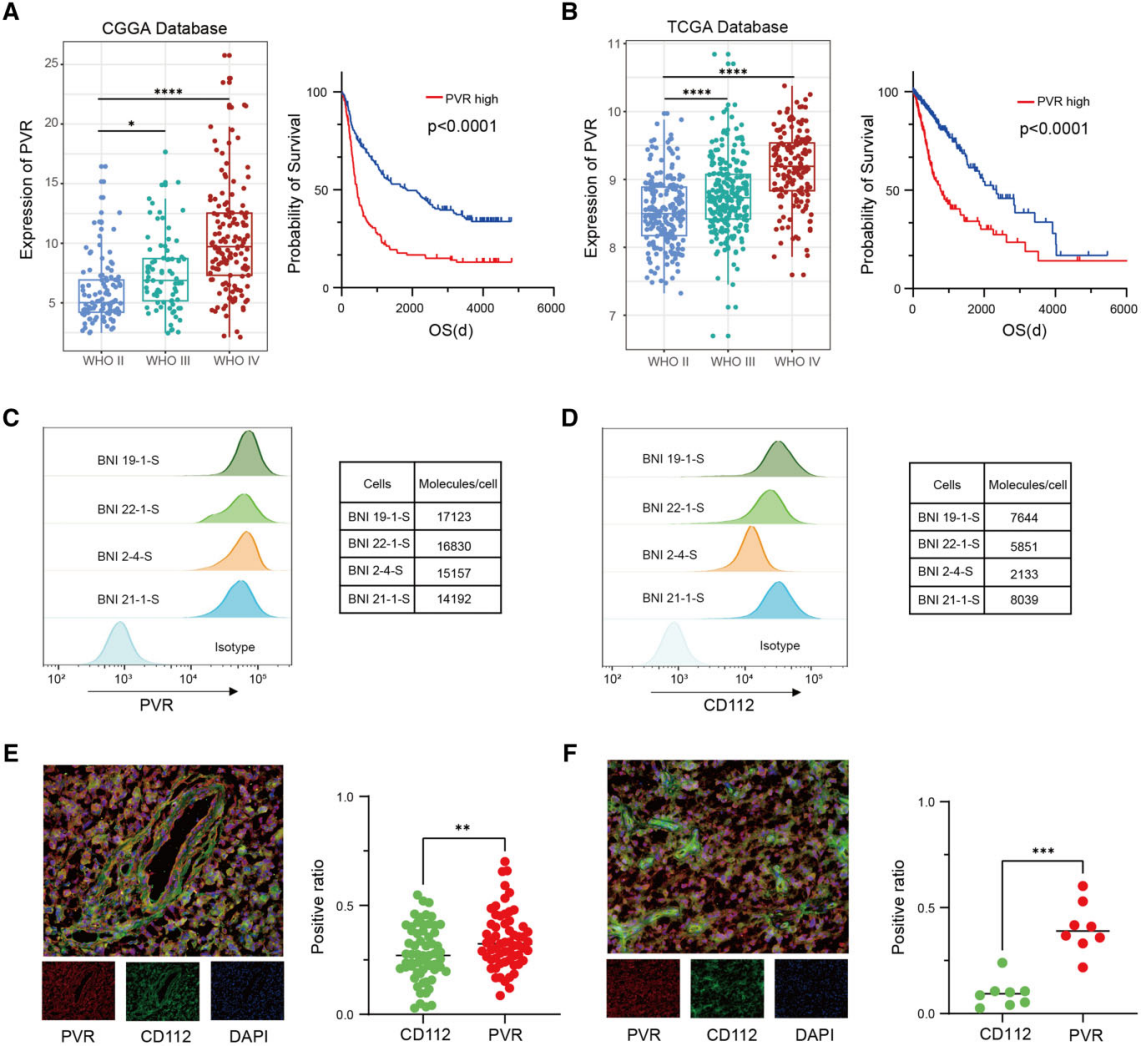
The expression of poliovirus receptor (PVR) in glioblastoma. **A, B**) The expression of PVR in glioma transcriptome sequencing data from the Chinese Glioma Genome Atlas (**A**) and The Cancer Genome Atlas (**B**) databases. **C, D**) Flow cytometric cell-surface expression of PVR (**C**) and CD112 (**D**) on 4 glioma stem cells. Tables show molecules per cell of PVR and CD112 as determined by a Quantibrite Phycoerythrin assay. **E, F**) Representative immunofluorescence images of PVR and CD112 in primary Isocitrate Dehydrogenase (IDH) wild-type glioblastoma (**E**) and recurrent IDH wild-type glioblastoma specimens (**F**). The ratios of positive staining were determined among primary IDH wild-type glioblastoma (n = 18) and recurrent IDH wild-type glioblastoma (n = 6). Scale bar: 100 μm. CCGA = Chinese Glioma Genome Atlas; OS = overall survival; TCGA = The Cancer Genome Atlas; WHO = World Health Organization. ****P < .0001, ***P< .001, **P < .01, *P < .05

We analyzed transcriptome sequencing databases of the Chinese Glioma Genome Atlas and The Cancer Genome Atlas and found poliovirus receptor to be highly expressed and positively correlated with glioma WHO grade and poorer prognosis (Figure 1, A and B). Poliovirus receptor expression was an independent prognostic factor on a multivariate cox analysis (Supplementary Tables 1 and 2, available online). Poliovirus receptor was also expressed in glioma stem cells at an average level of approximately 15 000 molecules per cell (Figure 1, C). Tyrosine-based inhibitory motif and DNAX accessory molecule 1 potentially interact with CD112 known as poliovirus receptor–related protein 2 (10), which was found to be expressed in glioma stem cells at a lower level compared with poliovirus receptor (Figure 1, D). Poliovirus receptor was also found to be expressed in other solid tumor cell lines, such as colorectal carcinoma and melanoma (Supplementary Figure 1, A, available online). K562 exhibited low poliovirus receptor expression, and almost no poliovirus receptor expression was detected in T cells (Supplementary Figure 1, B, available online).

These findings were supported by histological evaluation in samples from patients with primary and recurrent IDH wild-type glioblastoma and in organoids (Figure 1, E and F; Supplementary Figure 1, C, available online). We did not observe poliovirus receptor expression in normal brain tissues (Supplementary Figure 1, D, available online), in agreement with previous studies (4,5). Taken together, these results suggested that poliovirus receptor was a potential target for glioblastoma immunotherapy.

### Poliovirus receptor targeting chimeric antigen receptor T cells exhibit killing capacity against glioma stem cells

**Figure 2.**
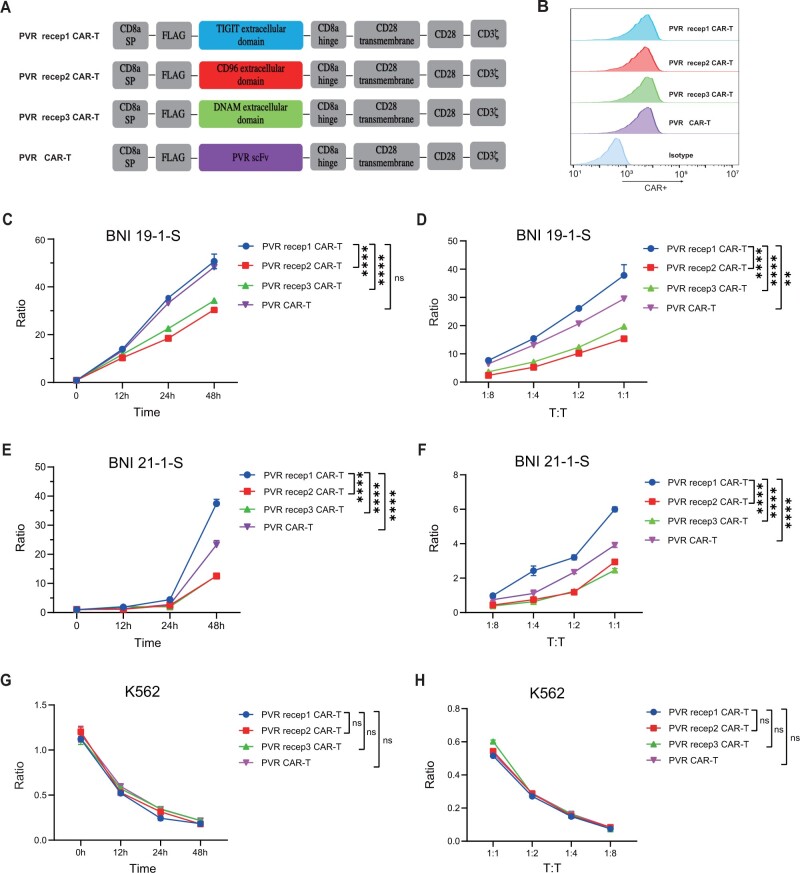
The construction of poliovirus receptor (PVR) targeting chimeric antigen receptor T (CAR-T) cells and efficacy against glioma stem cells. **A**) Schematic of CAR constructs. CAR molecules that employed the extracellular domains of TIGIT, CD96, and DNAM for recognizing PVR were called PVR receptor 1 CAR-T (PVR recep1 CAR-T), PVR receptor 2 CAR-T (PVR recep2 CAR-T), and PVR receptor 3 CAR-T (PVR recep3 CAR-T), respectively. The other CAR molecule employing the single-chain fragment variable of a PVR antibody was called PVR CAR-T cells. **B**) Four days after lentiviral transduction, transduction efficiency was measured by anti-DYKDDDDK Tag antibody using flow cytometry. **C, D**) CAR-T cells cocultured with BNI-19-1-S expressing fluorescent proteins at an Effector to Tumor ratio of 1:1 for 12 , 24,  and 48 hours (**C**) or at an Effector to Tumor ratio of 1:8, 1:4, 1:2, 1:1 for 24 hours (**D**). At each time point, E to T ratio was calculated to determine the killing capacity. **E, F**) CAR-T cells cocultured with BNI-21-1-S expressing fluorescent proteins at an Effector to Tumor ratio of 1:1 for 12, 24, and 48 hours (**E**) or at Effector to Tumor ratio of 1:8, 1:4, 1:2, 1:1 for 24 hours (**F**). **G, H**) CAR-T cells cocultured with K562 expressing fluorescent proteins at an Effector to Tumor ratio of 1:1 for 12, 24, and 48 hours (**G**) or at an Effector to Tumor ratio of 1:8, 1:4, 1:2, 1:1 for 24 hours (**H**). Data of in vitro killing are representative of 3 independent experiments. DNAM = DNAX accessory molecule-1; PVR recep1 CAR-T = poliovirus receptor 1 chimeric antigen receptor T cells; PVR recep2 CAR-T = poliovirus receptor 2 chimeric antigen receptor T cells; PVR recep3 CAR-T = poliovirus receptor 3 chimeric antigen receptor T cells; scFv = single-chain fragment variable; TIGIT = tyrosine-based inhibitory motif. ****P < .0001, ***P < .001, **P < .01, *P < 0.05, ^ns^*P* ≥ .05

Poliovirus receptor naturally interacts with tyrosine-based inhibitory motif, CD96, and DNAX accessory molecule 1 (10). We hypothesize that employing the extracellular domains of tyrosine-based inhibitory motif, CD96, and DNAX accessory molecule 1 is a potential strategy for chimeric antigen receptor molecules constructing against poliovirus receptor. Chimeric antigen receptor molecules that employed the extracellular domains of tyrosine-based inhibitory motif, CD96, and DNAX accessory molecule 1 were called poliovirus receptor 1 chimeric antigen receptor T cells, poliovirus receptor 2 chimeric antigen receptor T cells, and poliovirus receptor 3 chimeric antigen receptor T cells, respectively. The other chimeric antigen receptor molecule that employed the scFv of a poliovirus receptor antibody was called poliovirus receptor chimeric antigen receptor T cells (Figure 2, A). The 4 constructs exhibited similar expression of chimeric antigen receptor molecules and exhaustion markers (Figure 2, B; Supplementary Figure 2, A-C, available online). The majority of the chimeric antigen receptor T cells exhibited memory phenotypes (Supplementary Figure 2, D, available online). All sets of chimeric antigen receptor T cells had similar proliferation curve and could be activated by recombinant human poliovirus receptor protein (Supplementary Figure 2, E and F, available online).

Next, different chimeric antigen receptor T cells were cocultured with BNI-19-1-S cells. Poliovirus receptor 1 and poliovirus receptor chimeric antigen receptor T cells exhibited higher killing capacity than did the other 2 chimeric antigen receptor T cells (Figure 2, C). Poliovirus receptor 1 chimeric antigen receptor T cells could eradicate BNI-19-1-S cells quickly (Supplementary Video 1, available online). The efficacy of poliovirus receptor 1 chimeric antigen receptor T cells was more obvious in the presence of a higher number of target cells (Figure 2, D). When cocultured with BNI-21-1-S cells with lower poliovirus receptor expression, poliovirus receptor 1 chimeric antigen receptor T cells exhibited more obvious advantage at killing capacity compared with other chimeric antigen receptor T cells (Figure 2, E and F). Also, poliovirus receptor 1 chimeric antigen receptor T cells produced more tumor necrosis factor–⍺ (Supplementary Figure 2, G-N, available online). All chimeric antigen receptor T cells failed to suppress K562 with extremely low poliovirus receptor expression (Figure 2, G and H).

### Poliovirus receptor targeting chimeric antigen receptor T cells exerted antitumor effect in xenograft models with favorable safety profiles

**Figure 3.**
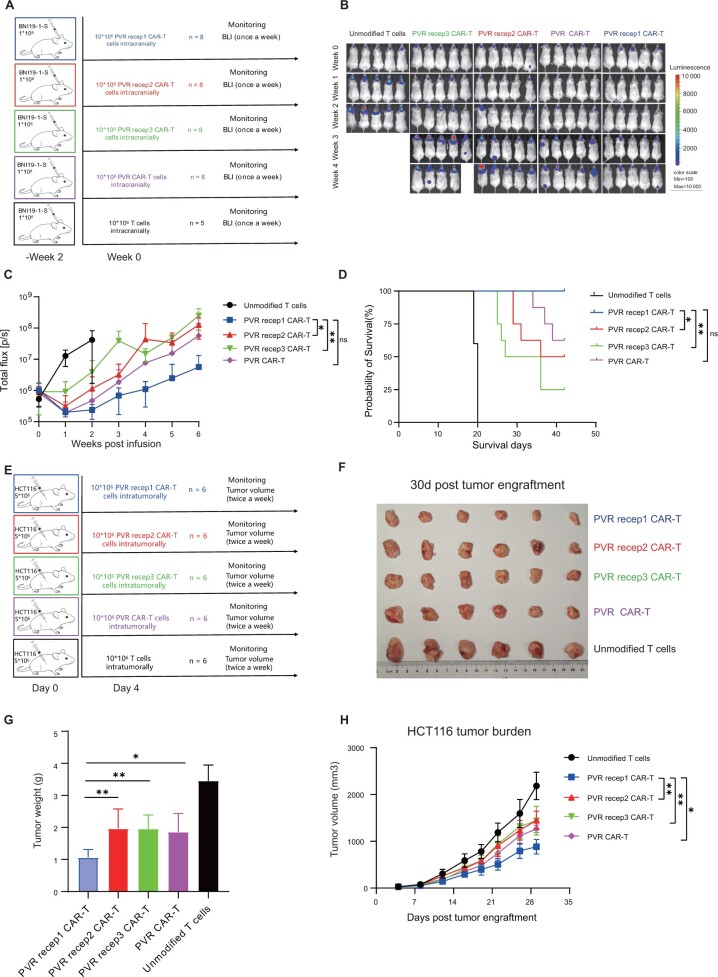
In vivo antitumor activity of poliovirus receptor–targeting chimeric antigen receptor T (CAR-T) cells. **A**) Schematic of the BNI-19-1-S orthotopic xenograft model. **B**) Bioluminescence imaging of tumor burden was assessed weekly by In Vivo Imaging System (IVIS) imaging. The data show the first 5 bioluminescence imaging. **C**) Quantification of bioluminescence imaging signal (unmodified T cells, n = 5; CAR-T cells, n = 8). **D**) Kaplan–Meier analysis of the outcome of CAR-T cells treatment. **E**) Schematic of colorectal carcinoma subcutaneous models using HCT116 cells. **F, G**) Images of tumor (**F**) and tumor weights (**G**) of mice at 30 days after tumor engraftment (n = 6 per group). **H**) Tumor volumes were assessed twice a week after tumor engraftment. Max = maximum; min = minimum; PVR = poliovirus receptor; PVR recep1 CAR-T = poliovirus receptor 1 chimeric antigen receptor T cells; PVR recep2 CAR-T = poliovirus receptor 2 chimeric antigen receptor T cells; PVR recep3 CAR-T = poliovirus receptor 3 chimeric antigen receptor T cells. ****P < .0001, ***P < .001, **P < .01, *P < .05, ^ns^*P* ≥ .05

Next, we evaluated the efficacy of chimeric antigen receptor T cells in an orthotopic tumor model using BNI-19-1-S (Figure 3, A). All chimeric antigen receptor T cells induced tumor regression within 1 week, and yet differences emerged by 3 weeks. The poliovirus receptor 1 chimeric antigen receptor T cells suppressed tumor growth most efficiently and achieved a survival benefit (Figure 3, B-D). To further assess the systemic toxicity of chimeric antigen receptor T cells, we evaluated the infiltration of chimeric antigen receptor T cells and found that no chimeric antigen receptor molecules or tissue-damaging effects were detected in the heart, liver, spleen, lung, and kidney (Supplementary Figure 3, A and B, available online). In addition, we monitored the body weights of mice twice per week after chimeric antigen receptor T cells administration, and no obvious treatment-associated weight loss was observed (Supplementary Figure 3, C, available online).

As noted above, poliovirus receptor was expressed in several solid tumors. We further investigated the antitumor effect of chimeric antigen receptor T cells in colorectal carcinoma subcutaneous model (Figure 3, E). Poliovirus receptor 1 chimeric antigen receptor T cells achieved the greatest tumor suppression (Figure 3, F-H). We also tested chimeric antigen receptor T cells in a melanoma xenograft model (Supplementary Figure 3, D, available online). Tumor burden was reduced by 7 days postadministration of chimeric antigen receptor T cells. Mice treated with poliovirus receptor 1 chimeric antigen receptor T cells achieved the greatest tumor suppression (Supplementary Figure 3, E-G, available online). Collectively, these data demonstrated that poliovirus receptor 1 chimeric antigen receptor T cells mediated higher and sustained antitumor effects compared with the counterpart constructs, and 4 chimeric antigen receptor T cells were associated with limited side effects.

### Poliovirus receptor 1 chimeric antigen receptor T cells demonstrated superior performance against glioma stem cells with lower poliovirus receptor expression

**Figure 4.**
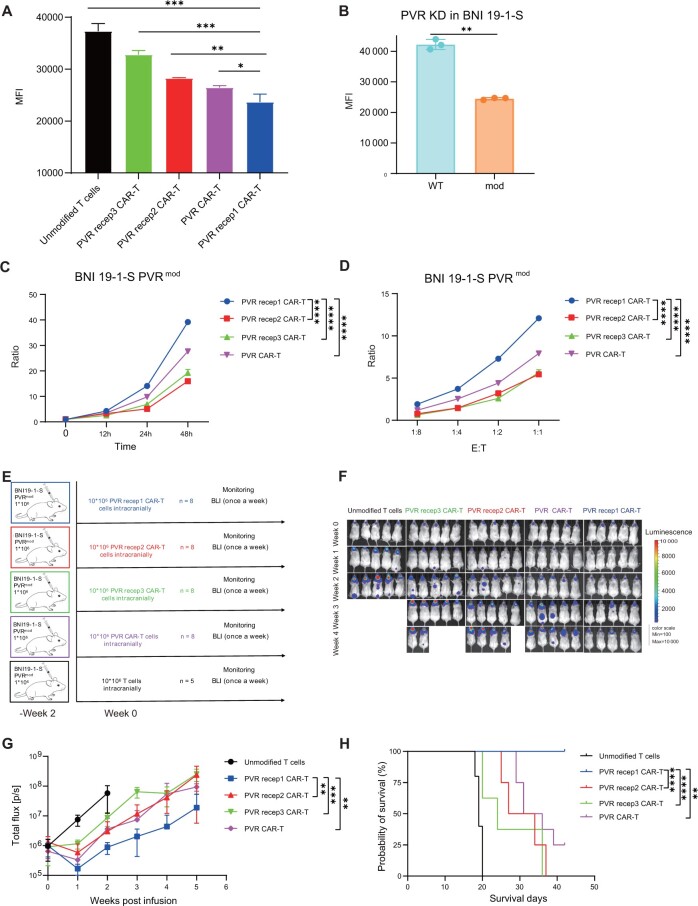
Antitumor activity of poliovirus receptor (PVR) targeting chimeric antigen receptor T (CAR-T) cells against glioma stem cells with lower PVR expression. **A**) Flow cytometric analysis of cell-surface expression of PVR on recurrent tumor cells. **B**) Knockdown PVR expression by small hairpin RNA (shRNA) and constructing BNI-19-1-S cells expressing moderate PVR (PVR^mod^). shRNA sequence: GGATCGGGATTTATTTCTATT. **C, D**) CAR-T cells cocultured with PVR^mod^ BNI-19-1-S expressing fluorescent proteins at an Effector to Tumor ratio of 1:1 for 12, 24, and 48 hours (**C**) or at an Effector to Tumor ratio of 1:8, 1:4, 1:2, 1:1 for 24 hours (**D**). At each time point, an E to T ratio was calculated to determine the killing capacity. Data are representative of 3 independent experiments. **E**) Schematic of PVR^mod^ BNI-19-1-S orthotopic xenograft model. **F**) Bioluminescence imaging of tumor burden was assessed weekly by In Vivo Imaging System (IVIS) imaging. The data show the first 5 bioluminescence imaging (BLI). **G**) Quantification of bioluminescence imaging signal (unmodified T cells, n = 5; CAR-T cells, n = 8). **H**) Kaplan–Meier analysis of the outcome of CAR-T cells treatment. Max = maximum; MFI = mean fluorescence intensity; Min = minimum; WT = wildtype; KD = knockdown; p/s = photons/sec; PVR recep1 CAR-T = poliovirus receptor 1 chimeric antigen receptor T cells; PVR recep2 CAR-T = poliovirus receptor 2 chimeric antigen receptor T cells; PVR recep3 CAR-T = poliovirus receptor 3 chimeric antigen receptor T cells. ****P < .0001, ***P < .001, **P < .01, *P < .05, ^ns^*P* ≥ .05

Tumor recurrence with decreased poliovirus receptor expression was observed in all chimeric antigen receptor T-cell treatment groups (Figure 4, A). The loss or downregulation of antigen has emerged as a common mechanism of resistance to chimeric antigen receptor T-cell therapeutics (20). Therefore, antigen escape may drive the decreasing effect of poliovirus receptor targeting chimeric antigen receptor T cells. To evaluate which chimeric antigen receptor T cell was more tolerant to antigen loss, we knocked down poliovirus receptor expression in BNI-19-1-S and constructed cells expressing moderate (approximately half of the parent) poliovirus receptor antigen (Figure 4, B). Poliovirus receptor 1 chimeric antigen receptor T cells exhibited superior performance against poliovirus receptor moderate BNI-19-1-S cells compared with other chimeric antigen receptor T cells (Figure 4, C and D). Also, poliovirus receptor 1 chimeric antigen receptor T cells produced more cytokines including grazyme A, grazyme B, interferon-γ, and tumor necrosis factor–⍺ (Supplementary Figure 4, A-D, available online). In vivo orthotopic models also indicated that poliovirus receptor 1 chimeric antigen receptor T cells achieved the superior antitumor effect and survival (Figure 4, E-H). These results demonstrated that poliovirus receptor 1 chimeric antigen receptor T cell was more tolerant to antigen loss.

### Poliovirus receptor 1 chimeric antigen receptor T cells failed to control glioma stem cells with low poliovirus receptor expression

**Figure 5.**
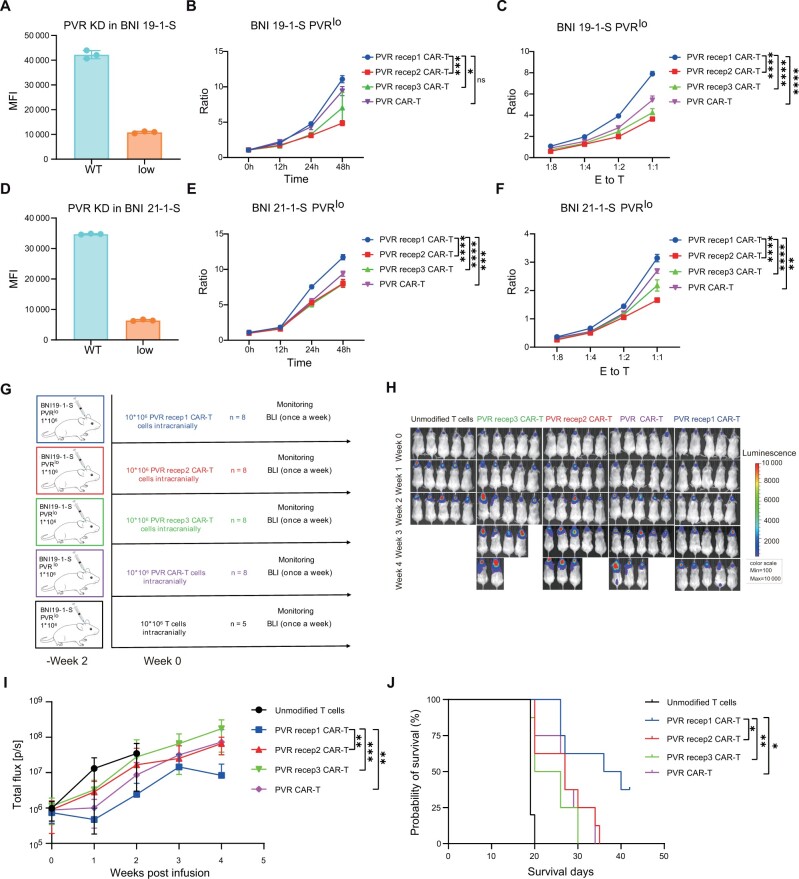
Antitumor activity of poliovirus receptor (PVR) targeting chimeric antigen receptor T (CAR-T) against glioma stem cells with low PVR expression. **A**) Knockdown PVR expression by small hairpin RNA (shRNA) and constructing BNI-19-1-S cells expressing low PVR (PVR^lo^). shRNA sequence: GGATCGGGATTTATTTCTATT. **B, C**) CAR-T cells cocultured with PVR^lo^ BNI-19-1-S expressing fluorescent proteins at an Effector to Tumor ratio of 1:1 for 12, 24, and 48 hours (**B**) or at an Effector to Tumor ratio of 1:8, 1:4, 1:2, 1:1 for 24 hours (**C**). At each time point, an Effector to Tumor ratio was calculated to determine the killing capacity. **D**) Knockdown PVR expression by shRNA and constructing BNI-21-1-S cells expressing low PVR. shRNA sequence: GGATCGGGATTTATTTCTATT. **E, F**) CAR-T cells cocultured with PVR^lo^ BNI-21-1-S expressing fluorescent proteins at an Effector to Tumor ratio of 1:1 for 12, 24, and 48 hours (**E**) or at an Effector to Tumor ratio of 1:8, 1:4, 1:2, 1:1 for 24 hours (**F**). At each time point, an Effector to Tumor ratio was calculated to determine the killing capacity. Data of in vitro killing are representative of 3 independent experiments. **G**) Schematic of the PVR^lo^ BNI-19-1-S orthotopic xenograft model. **H**) Bioluminescence imaging (BLI) of tumor burden was assessed weekly by In Vivo Imaging System (IVIS) imaging. **I**) Quantification of bioluminescence imaging signal (unmodified T cells, n = 5; CAR-T cells, n = 8). **J**) Kaplan–Meier analysis of the outcome of CAR-T cells treatment. Max = maximum; MFI = mean fluorescence intensity; Min = minimum; PVR recep1 CAR-T = poliovirus receptor 1 chimeric antigen receptor T cells; PVR recep2 CAR-T = poliovirus receptor 2 chimeric antigen receptor T cells; PVR recep3 CAR-T = poliovirus receptor 3 chimeric antigen receptor T cells.

The duration of tumor regression exerted by poliovirus receptor 1 chimeric antigen receptor T cells was shortened in mice modeled with poliovirus receptor^mod^ BNI-19-1-S cells as compared with normal BNI-19-1-S. To further investigate the extent to which poliovirus receptor 1 chimeric antigen receptor T cells could tolerate antigen escape, we constructed BNI-19-1-S and BNI-21-1-S with low poliovirus receptor expression (Figure 5, A and D). Although poliovirus receptor 1 chimeric antigen receptor T cells had stronger antigen sensitivity than other chimeric antigen receptor T cells, it retained only a limited killing effect on glioma stem cells with very low antigen density (Figure 5, B and C, E and F). The secretion of most cytokines also decreases substantially compared with coculturing with parental glioma stem cells (Supplementary Figure 4, E-L, available online). Accordingly, in vivo poliovirus receptor low BNI-19-1-S orthotopic models also demonstrated an earlier and faster relapse (Figure 5, G-J). Together, these data confirmed that the cytotoxicity of poliovirus receptor 1 chimeric antigen receptor T cells required a certain antigen density threshold.

### Tumor recurrence can be abrogated by sequential therapy combining poliovirus receptor 1 chimeric antigen receptor T cells and NK-92 cells

**Figure 6.**
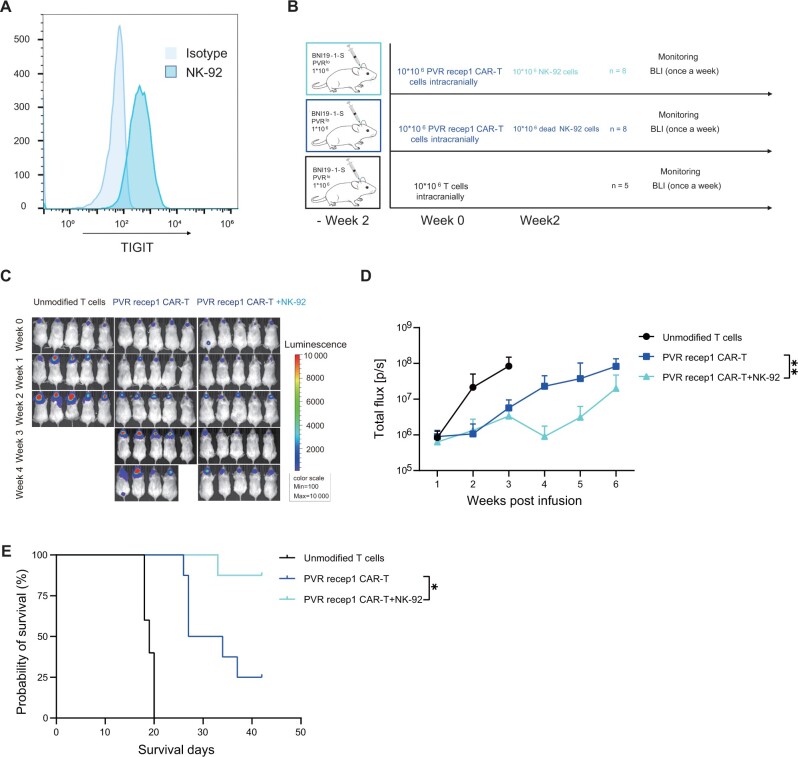
Sequential therapy combining poliovirus receptor (PVR) 1 chimeric antigen receptor T (CAR-T) cells and NK-92 abrogates tumor recurrence. **A**) Flow cytometric analysis of the expression of tyrosine-based inhibitory motif on the surface of NK-92 cells. **B**) Schematic of the PVR^lo^ BNI-19-1-S orthotopic xenograft model. **C**) Bioluminescence imaging (BLI) of tumor burden was assessed weekly by In Vivo Imaging System (IVIS) imaging. The data show the first 5 bioluminescence imaging. **D**) Quantification of bioluminescence imaging signal (unmodified T cells, n = 5; CAR-T cells, n = 8). **E**) Kaplan–Meier analysis of the outcome of CAR-T cells treatment. Max = maximum; Min = minimum; PVR recep1 CAR-T = poliovirus receptor 1 chimeric antigen receptor T cells; PVR recep2 CAR-T = poliovirus receptor 2 chimeric antigen receptor T cells; PVR recep3 CAR-T = poliovirus receptor 3 chimeric antigen receptor T cells; TIGIT = tyrosine-based inhibitory motif.

To improve the poor efficacy of chimeric antigen receptor T cells against glioma stem cells with low antigen density, we explored combinations of therapies. Glioma has been reported to constitutively express the NKG2D ligands including major histocompatibility complex (MHC) class I chain-related molecules A and B and members of the UL16-binding protein family (21). These results were supported by analysis of the CCGA and TGGA databases (Supplementary Figure 5, A and B, available online). Although these molecules are expected to activate NK cells, glioma cells could still resist NK cell killing because of high expression of MHC class I molecules (Supplementary Figure 5, C and D, available online). NK-92 cells have been shown to lack almost all killer cell immunoglobulin-like receptors that mediate inhibitory effects by binding to allotypic determinants that are shared by MHC class I molecules (22,23). NK-92 cells were found to express high levels of tyrosine-based inhibitory motif at steady state (Figure 6, A). Therefore, we hypothesized that NK-92 cells might be easier to be inhibited in poliovirus receptor high tumors but would be more effective in tumors with low poliovirus receptor expression.

To address this issue, we assessed the antitumor efficacy of a sequential therapy combining poliovirus receptor 1 chimeric antigen receptor T cells and NK-92 cells in a poliovirus receptor low BNI-19-1-S orthotopic model. NK-92 cells were administrated intratumorally 2 weeks after poliovirus receptor 1 chimeric antigen receptor T-cell infusion (Figure 6, B). Tumor regression was found to be stronger and more durable as compared with monotherapy with poliovirus receptor 1 chimeric antigen receptor T cells (Figure 6, C-E). Thus, sequential therapy using poliovirus receptor 1 chimeric antigen receptor T cells and NK-92 cells is a potential strategy to overcome tumor recurrence because of antigen loss.

## Discussion

Poliovirus receptor overexpression correlates with tumor progression and poor prognosis in various cancers (15). Therapies targeting poliovirus receptor and poliovirus receptor–like proteins, including oncolytic polio virotherapy and antibodies, have been tested (10). Here, we developed poliovirus receptor–based chimeric antigen receptor T cells and a classical chimeric antigen receptor T cell using the scFv.

Because poliovirus receptor and CD112 can interact with poliovirus receptor–like proteins including tyrosine-based inhibitory motif, CD96, and DNAX accessory molecule-1, we first evaluated the cytotoxicity of chimeric antigen receptor T cells against 2 cells lines, BNI-19-1-S and BNI-21-1-S, that have different levels of poliovirus receptor expression but equal expression of CD112 to exclude interference of CD112. Among the 4 chimeric antigen receptor constructs, poliovirus receptor 1 chimeric antigen receptor T cells exhibited higher cytotoxicity. Poliovirus receptor 1 chimeric antigen receptor T cells suppressed tumor growth efficiently in an orthotopic xenograft model. We also demonstrated the antitumor effect of these chimeric antigen receptor T cells in melanoma and colorectal subcutaneous models, indicating that poliovirus receptor targeting chimeric antigen receptor T cells might also serve as a potential therapy for other solid tumors that overexpress poliovirus receptor.

We noticed a recurrence of tumors upon long-term treatment with poliovirus receptor targeting chimeric antigen receptor T cells. A common mechanism of resistance to chimeric antigen receptor T-cell therapy is the emergence of antigen loss. For example, relapse related to the loss of CD19 had been frequently reported in chimeric antigen receptor T-cell treatments of acute lymphoblastic leukemia (24-26). The loss of estimated glomerular filtration rate (EGFR) vIII and interleukin (IL)13Rα2 has also been observed in chimeric antigen receptor T-cell–treated patients with IDH wild-type glioblastoma (27,28). Our data also suggested the same phenomenon that recurred tumor had decreased poliovirus receptor expression.

Two key approaches are usually adopted to overcome antigen loss: modifying chimeric antigen receptors to increase antigen sensitivity (29,30) and simultaneously targeting 2 or more tumor cell antigens (31-33). For example, chimeric antigen receptor T cells simultaneously targeting HER2, IL13Rα2, and ephrin-A2 have been reported to overcome antigenic heterogeneity and lead to improved treatment outcomes in IDH wild-type glioblastoma (34,35). However, in our research, we chose to use NK-92 cells in combination. NK cell function is regulated by an array of activating and inhibitory receptors (36). Activating receptors, mainly including CD16 and NKG2D, can mediate antitumor effects (37-39). Inhibitory receptors mainly include the killer cell immunoglobulin-like receptors. Killer cell immunoglobulin-like receptors protect healthy autologous cells from attack on interaction with autologous MHC class I molecules (40), which can also limit the application of NK cells to antitumor treatments. NK-92 cells lack almost all killer cell immunoglobulin-like receptors (22,23). Meanwhile, the high expression of the NKG2D ligands was validated in glioma. Our data also suggested NK-92 expressed high level of tyrosine-based inhibitory motif that has recently emerged as an inhibitory checkpoint that could suppress functions of T cells and NK cells by binding to poliovirus receptor. Therefore, NK-92 cells would be more effective in tumors with low poliovirus receptor expression. As expected, combinational therapy using poliovirus receptor 1 chimeric antigen receptor T cells and NK-92 cells substantially prolonged survival and overcame tumor recurrence.

In addition, chimeric antigen receptor NK-92 targeting poliovirus receptor could be a potential strategy in IDH wild-type glioblastoma treatment. Chimeric antigen receptor NK cells can mediate cytotoxic activity against tumors in chimeric antigen receptor–dependent and chimeric antigen receptor–independent manners. NK-92 cell line is a potential alternative candidate for chimeric antigen receptor NK immunotherapy (41). At present, chimeric antigen receptor NK-92 targeting HER2 (42,43), wild-type EGFR and EGFRvIII (44,45), and GD2 and NKG2D ligands (46) have been tested in previous studies. These chimeric antigen receptor NK-92 cells displayed enhanced cytolytic capability and resulted in efficient suppression of tumor growth in glioblastoma xenograft mouse models. A multicenter, open label, phase I study reported that intracranial injection of HER2-targeted chimeric antigen receptor NK92 cells was feasible and safe in patients with recurrent HER2-positive IDH wild-type glioblastoma (47). However, these chimeric antigen receptor constructs mostly adopted the second generation of chimeric antigen receptor T cells incorporating CD28-CD3ζ signaling domain (43). The chimeric antigen receptor constructure is becoming increasingly sophisticated with the understanding of NK cell activation and tumor-specific or -associated antigens. It is feasible and potential to design chimeric antigen receptors specific to NK cells. The construct, incorporating the transmembrane of NKG2D and intracellular domains of 2B4, has been validated to exhibit-superior in vitro and in vivo antitumor activities compared with second-generation chimeric antigen receptor T cells (48).

In theory, T cells and NK cells have a synergistic effect in tumor eradication. CD8 T cells kill cancer cells by T-cell receptor recognition of tumor cell–derived peptides and MHC class I protein complex (49). Tumor cells could escape the attack by loss of MHC class I expression (50), which sensitizes tumor cells to be killed by NK cells (40). Several other mechanisms about the synergistic effects have been found. NK cells secrete chemokines to recruit and support the survival of dendritic cells (51,52), which is essential for antitumor immunity mediated by T cells (53). NK cells were found to enhance chimeric antigen receptor T-cell antitumor efficacy by enhancing immune-tumor cell cluster formation and preventing exhaustion and senescence of T cells (54). Another study reported that chimeric antigen receptor NK cells could target and eliminate myeloid-derived suppressor cells and improve the infiltration and functions of subsequently infused chimeric antigen receptor T cells by secreting proinflammatory cytokines and chemokines (55). Therefore, immunotherapy-combined T cells with NK cells is a potential strategy for tumor treatment.

In summary, we reported the development of a poliovirus receptor targeting chimeric antigen receptor T-cell treatment strategy for IDH wild-type glioblastoma. In vivo and in vitro experiments demonstrated a robust cytotoxicity exerted by chimeric antigen receptor T cells against glioma stem cells and other solid tumors with an acceptable safety profile. Poliovirus receptor 1 chimeric antigen receptor T cells were most tolerant to tumor antigen escape. A combinational therapy of poliovirus receptor 1 chimeric antigen receptor-T cells and NK-92 cells markedly prolonged survival and overcame tumor recurrence.

## Supplementary Material

djad226_Supplementary_Data

## Data Availability

The transcriptome sequencing data of IDH wild-type glioblastoma are publicly available in the portal sites of the CGGA (http://www.cgga.org.cn/) and TCGA (https://portal.gdc.cancer.gov). The data generated in this study are available upon request from the corresponding author.
