# Supplementary material for: Poliovirus receptor–based chimeric antigen receptor T cells combined with NK-92 cells exert potent activity against glioblastoma
Source: J Natl Cancer Inst. 2023 Nov 7;116(3):389–400. doi: 10.1093/jnci/djad226 (PMC10919341; doi:10.1093/jnci/djad226)
Supplement: djad226_Supplementary_Data [file djad226_supplementary_data.zip › djad226_Supplementary_Data/Supplemental Materials 10.31.23.pdf]

Supplementary Methods

Primary Cells and Cell Lines

All glioma stem cells (BNI-19-1-S, BNI-21-1-S, BNI-22-1-S, and BNI-2-4-S) were constructed in our laboratory. Briefly, tumor tissues obtained during surgery were immediately immersed in the medium and transported to the laboratory on ice for further processing. The tissue was cleaned and shredded mechanically and then enzymatically digested into single cells using trypsin. The single cells were filtered using a 200-mesh filter and centrifuged (400 g) for 5 min. After treating the cells with red blood cell lysis, they were centrifuged again. The obtained cells were cultured in a serum-free medium containing DMEM/F12 (Gibco) supplemented with B27 (Gibco), basic fibroblast growth factor (bFGF, 20 ng/mL), epidermal growth factor (EGF, 20 ng/mL), and heparin (2.5 mg/mL). Primary GSCs were enzymatically dissociated into single cells using Accutase (Sigma Aldrich) and thereafter routinely cultured in the serum-free medium that was replaced every 4–6 days. U87, A375, HCT116, and LS174T cell lines were cultured in DMEM supplemented with 10% FBS and 1% penicillin-streptomycin (Thermo Fisher Scientific). MDA-MB-453 cells were cultured in Leibovitz's L-15 supplemented with 10% FBS and 1% penicillin-streptomycin. K562 cells were a gift from the National Clinical Research Center for Blood Diseases, Chinese Academy of Medical Sciences and Peking Union Medical College and was cultured in RPMI1640 supplemented with 10% FBS and 1% penicillin-streptomycin. BNI-19-1-S, BNI-21-1-S, and K562 cells were transduced to express green fluorescent protein (GFP) and BNI-19-1-S cells were also transduced to express

luciferase. The expression of PVR was knocked down in BNI-19-1-S and BNI-21-1-S cells using the shRNA GGATCGGGATTTATTTCTATT. All transduced cell lines were selected in the presence of puromycin or neomycin (Solarbio, 5 μg/mL) for at least 2 weeks.

Generation of Glioblastoma Organoids (GBO)

Fresh surgical specimens were minced and cultured in 6 well plates containing 3 mL of GBO medium (50% DMEM/F12 (Gibco), 50% Neurobasal (Gibco), 1% GlutaMax (Gibco), 1% non-essential amino acids (Gibco), 1% penicillin-streptomycin (Thermo Fisher Scientific), 1% N2 supplement (Thermo Fisher Scientific), and B27), and placed on an orbital shaker rotating at 140 rpm in a sterile 37 ºC incubator with an atmosphere of 5% CO_2 and 90% humidity. Within 2 to 3 weeks, tumour pieces generally formed rounded organoids that were ready to be further tested.

Tumor Antigen Density Quantification

Cells were harvested in the logarithmic phase and analyzed by flow cytometry using a phycoerythrin (PE)-labelled PVR antibody (Biolegend). Antigen density was calculated using the BD Quantibrite™ PE (Cat.No. 340495) according to the manufacturer's instructions. Briefly, BD Quantibrite™ PE tube contains a lyophilized pellet of beads that are pre-calibrated standard bead sets containing known numbers of fluorophore molecules. When we run a BD Quantibrite™ PE tube by flow cytometer, we can plot a linear regression of Log10 PE molecules per bead against Log10 fluorescence, using the following equation: $y = mx + c$, where y equals Log10

fluorescence and x equals Log10 PE molecules per bead. When we run an unknown cell population at the same flow cytometer, fluorescence signal can be converted into number of fluorophores. They allow for calculation of antigen molecules number per cell when we know the corresponding fluorophores to protein ratio (F:P) of each antibody. Information on F:P ratio of each antibody was provided by Biolegend for CD112-PE-TX31 (Cat#337409=1:1) and PVR-PE-TX24 (Cat#337507=1:1).

Plasmid and Lentivirus Production

The CAR constructs used in this study contained a CD8α leader sequence, 3× FLAG tags, a CD8 hinge domain, a CD28 transmembrane domain, and CD3ζ and CD28 intracellular domains. The scFv of an anti-PVR antibody (US20210115150A1), or the extracellular domain of human TIGIT, CD96 or DNAM was inserted between the FLAG sequences and the CD8α hinge domain to generate the corresponding CAR gene. CAR expression was detected using an anti-FLAG antibody. The lentiviral products were purchased from Beijing SyngenTech Co., LTD.

T Cells Isolation and CAR-T Cells Production

Peripheral blood mononuclear cells (PBMCs) were isolated from blood via density gradient centrifugation. T cells were purified from PBMCs using a human CD3 T cell isolation kit (Stemcell Technology). Isolated T cells were activated by T Cell TransAct^{TM} (Miltenyi Biotec). 48 hours after activation, lentivirus was loaded onto Retronectin (Takara) pre-coated non-tissue culture plates. Next, the activated T cells were added to the plates and centrifuged at 2000*g* for 90 min. During the entire

process, the cells were cultured in ImmunoCult™-XF T Cell Expansion Medium (Stemcell Technology), supplemented with 5 ng/mL IL-15 (Peprotech) and 10 ng/mL IL-7 (Peprotech). CAR-T cells were expanded until 8 days post-activation for experiments.

Antibodies and Flow Cytometry Analysis

Monoclonal antibodies compatible with flow cytometry (BioLegend) were used according to the manufacturer's instructions. Antibodies included allophycocyanin (APC)-conjugated anti-PD-1 and anti-TIM3; PE-conjugated anti-CTLA-4, anti-CD62L, anti-TIGIT, anti-CD112, anti-PVR and anti-CD107a; and fluorescein isothiocyanate (FITC)-conjugated anti-CD45RA. Cells were collected, washed, and stained with antibodies for 30 min at 4 °C in the dark and then analyzed with a flow cytometer (Accuri C6 Plus, BD). Transduced peripheral blood T cells were incubated with PE-conjugated anti FLAG antibody (BioLegend) to detect CAR expression.

Flow Cytometric Calcium Assay.

CAR-T cells (2×10^6) were washed and resuspended in 200 μL assay buffer. 2 μL Calbryte™ 520 AM stock solution was added into another 200 μL assay buffer and incubated with CAR-T cells at 37 °C for 20 minutes. The sample was then analysed on a flow cytometer using the FITC channel. At 2 minutes, an agonist (phorbol myristate acetate) or recombinant PVR protein was added to the sample; the entire analysis process lasted for 15 minutes.

Proliferation Assays

For the quantification of T cell expansion, CAR-T cells were cultured in 6-well plates using fresh ImmunoCult™-XF T Cell Expansion Medium supplemented with IL-15 and IL-7. The cells were quantified with an Automatic Cell Fluorescence Analyzer (shanghai Ruiyu Biotech, Co., Ltd, RY122F2017) every 2 days after lentivirus transduction.

Multiplex Cytokine Release Assay

CAR-T cells were co-cultured with tumour cells at an E:T ratio of 1:1 in ImmunoCult™-XF T Cell Expansion medium without additional cytokines supplementation. After 24 h of incubation, the supernatant was collected and analyzed by the Human Premixed Multi-Analyte kit (LXSAHM-05) on a Luminex 200 system (Luminex Corporation, Austin, TX, USA) according to manufacturer's protocol.

Immunofluorescence

Glioblastoma organoids were harvested and fixed for 15 minutes in 4% paraformaldehyde and dehydrated in 30% sucrose for 2 d. Serial 10 μm sections were cut on a cryotome (REM-710; Yamato). Non-specific binding sites on the sections were blocked and then stained for 2 h at 37 °C with anti-PVR (Proteintech) and anti-CD112 (Proteintech) antibodies, diluted 1:200. The sections were washed and then incubated with Alexa Fluor-488 or -594-conjugated secondary antibodies (1:500, Abcam) at 37 °C for 30 minutes and counterstained with DAPI (Solarbio). Mounted samples were imaged by microscopy (Observer Z1 and Axio Scan Z1, ZEISS). Immuno-positive cells were quantified manually using ImageJ (v1.51).

Hematoxylin-Eosin (HE) Staining

The organs of mice were removed after transcardial perfusion with 4% paraformaldehyde. Samples were fixed overnight in 4% paraformaldehyde followed by automatic dehydration and embedding in OCT compound. After freezing at -80°C, serial 5 μm (spleen) or 10 μm (heart, lung, liver and kidney) sections were prepared using a microtome (REM-710; Yamato). Sections were stained following standard HE protocols.

***In Vitro* Tracing of CAR-T-GSC Sphere Interactions**

BNI-19-1-S spheres were harvested at logarithmic phase. Half of the harvested spheres were digested in Accutase (Millipore) for automated cell counting (Millipore). The rest of the harvested spheres were stained with Cell Explorer™ Live Cell Tracking Kit *Orange Fluorescence* (AAT Bioquest) following the manufacturer's instructions. PVR recep1 CAR-T cells were stained with PKH67 Green Fluorescent Cell Linker Mini Kit (Sigma-Aldrich) following the manufacturer's instruction. Cells were co-cultured in ImmunoCult™-XF T Cell Expansion Medium at E:T ratio of 1:1. The mixture was suspended in a confocal dish and placed in Live Cell Station (Zeiss Axio Observer Z1). Images were taken every 5 minutes.

***In Vivo* Tumor Modelling and CAR-T Therapy**

All mice were housed in specific pathogen-free conditions at a barrier facility at Beijing Tiantan Hospital. All mice handling, surveillance and experimentation was performed in accordance with guidelines and approval from the Laboratory Animal

Care facility of Beijing Tiantan Hospital (IRB, ID:201904005). Mice in each room were observed daily by the animal technician for signs of illness.

Orthotopic Tumor Modelling

Briefly, mice were anaesthetised with 3% isoflurane in an induction chamber, and anaesthesia was maintained with application of 2% isoflurane through a nose adapter. A burr hole was placed 2 mm lateral and 1 mm anterior of the bregma. A blunt-ended needle (75 N, 26 s gauge, 2 in, point style 2, 5 μL; Hamilton Company) was lowered into the burr hole to a depth of 3.5 mm below the dura. Using a microinjection pump, an aliquot (5 μL) of 1×10^6 BNI-19-1-S luciferase cells suspended in PBS was injected within 5 minutes, and the needle was left in place for 1 minute following injection. Tumour growth was monitored by bioluminescence on an IVIS Spectrum In Vivo Imaging System and quantified with Live Image software (v 4.0; Living Image; PerkinElmer). After confirmation of tumour engraftment, the mice were divided into several treatment groups based on flux values. A single sample of 10×10^6 CAR-T cells or 10×10^6 T cells in 10 μL DPBS (Gibco) was injected into the predefined coordinates (2 mm lateral and 1 mm anterior of bregma) using the same stereotactic equipment and anaesthesia. Bodyweights of mice were determined before injection of CAR-T cells or unmodified T cells.

Subcutaneous Tumor Modelling

To produce subcutaneous models, male NSG mice aged 8 weeks were challenged with a subcutaneous injection of 5×10^6 A375 or HCT116 tumour cells. Tumour

volumes were measured using Vernier callipers according to the following formula: volume = (length × width^2)/2. Once the tumours reached a volume of 30 to 60 mm^3, mice were distributed into groups and treated with a single intratumoral injection of 10×10^6 CAR-T cells or 10×10^6 T cells in 50 μL DPBS. Tumour volumes were calculated twice per week, and when the maximal tumour burden was reached, the animal was sacrificed.

Supplementary Table 1. Univariate and multivariate analysis of prognostic parameters in CGGA database (OS)

Variable	Univariate analysis		Multivariate analysis	
	HR (95% CI)	P-Value	HR (95% CI)	P-Value
**Histology (WHO CNS 5th)** [a]				
Astrocytoma vs Glioblastoma	0.081 (0.046-0.142)	< 0.0001	0.112 (0.060-0.210)	< 0.0001
Oligodendroglioma vs Glioblastoma	0.395 (0.282-0.553)	< 0.0001	0.498 (0.331-0.750)	0.0008
**Age at Diagnosis**				
One-year older	1.031 (1.016-1.046)	< 0.0001	1.000 (0.984-1.017)	0.9600
**MGMT Status**				
Methy vs Un-Methy	0.864 (0.639-1.169)	0.3442		
**PVR Expression**				
High vs Low	3.293 (2.278-4.760)	< 0.0001	1.915 (1.281-2.865)	0.0016

[a] Histology refers to WHO Classification of Central Nervous System Tumor, the Fifth Edition. CI = confidence interval; HR = hazard ratio; Astrocytoma = Astrocytoma, IDH-mutant; Oligodendroglioma = Oligodendroglioma, IDH-mutant and 1p/19q-codeleted; Glioblastoma = Glioblastoma, IDH-wildtype.

Supplementary Table 2. Univariate and multivariate analysis of prognostic parameters in TGGA database (OS)

Variable	Univariate analysis		Multivariate analysis	
	HR (95% CI)	P-Value	HR (95% CI)	P-Value
**Histology (WHO CNS 5th)** [a]				
Astrocytoma vs Glioblastoma	0.028 (0.014-0.055)	< 0.0001	0.071 (0.033-0.152)	< 0.0001
Oligodendroglioma vs Glioblastoma	0.039 (0.022-0.070)	< 0.0001	0.127 (0.062-0.263)	< 0.0001
**Age at Diagnosis**				
One-year older	1.085 (1.068-1.103)	< 0.0001	1.050 (1.029-1.070)	< 0.0001
**MGMT Status**				
Methy vs Un-Methy	0.208 (0.139-0.313)	< 0.0001	0.593 (0.361-0.974)	0.0392
**PVR Expression**				
High vs Low	18.082 (5.489-59.571)	< 0.0001	3.674 (1.066-12.658)	0.0392

[a] Histology refers to WHO Classification of Central Nervous System Tumor, the Fifth Edition. CI = confidence interval; HR = hazard ratio; Astrocytoma = Astrocytoma, IDH-mutant; Oligodendroglioma = Oligodendroglioma, IDH-mutant and 1p/19q-codeleted; Glioblastoma = Glioblastoma, IDH-wildtype.

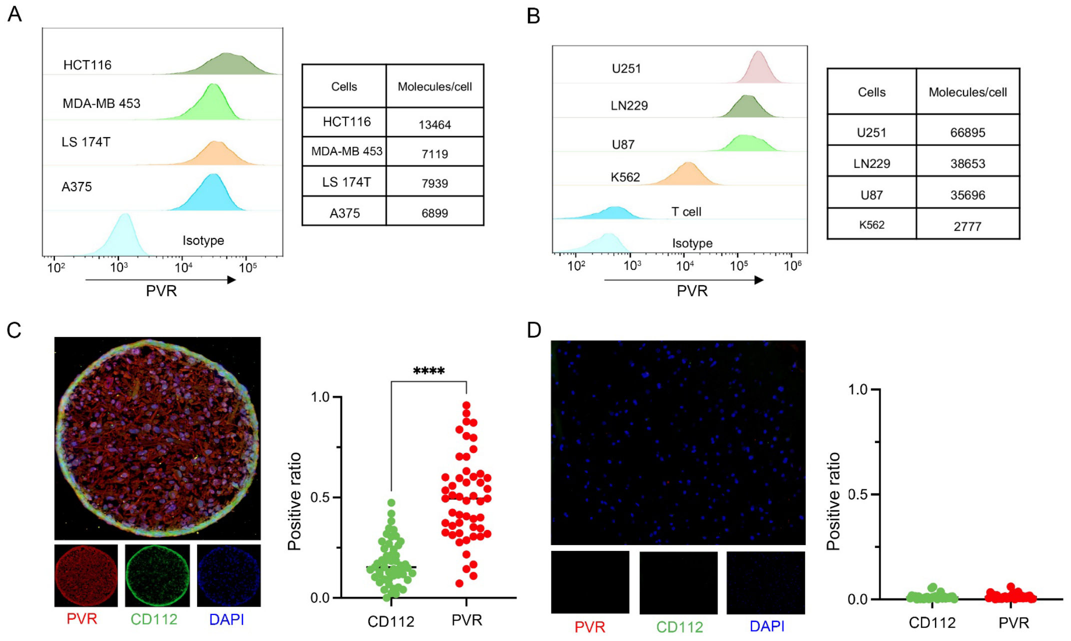

Supplementary Figure 1. The expression of PVR and CD112 in cell lines and specimens. A) Flow cytometric analysis of the expression of PVR on solid tumour cells: HCT116, colorectal carcinoma; MDA-MB-453, breast cancer; LS174T, colorectal carcinoma; A375, melanoma. The table shows molecules of PVR per cell as determined by a Quantibrite™ PE assay. **B**) Flow cytometric analysis of the expression of PVR on U87, LN229, U251, K562 and T cells. **C, D**) Representative immunofluorescence images of PVR and CD112 in glioblastoma organoids (**C**) and normal brain specimens (**D**), glioblastoma organoids (n = 16) and normal brain specimens (n = 7). Scale bar: 100µm.

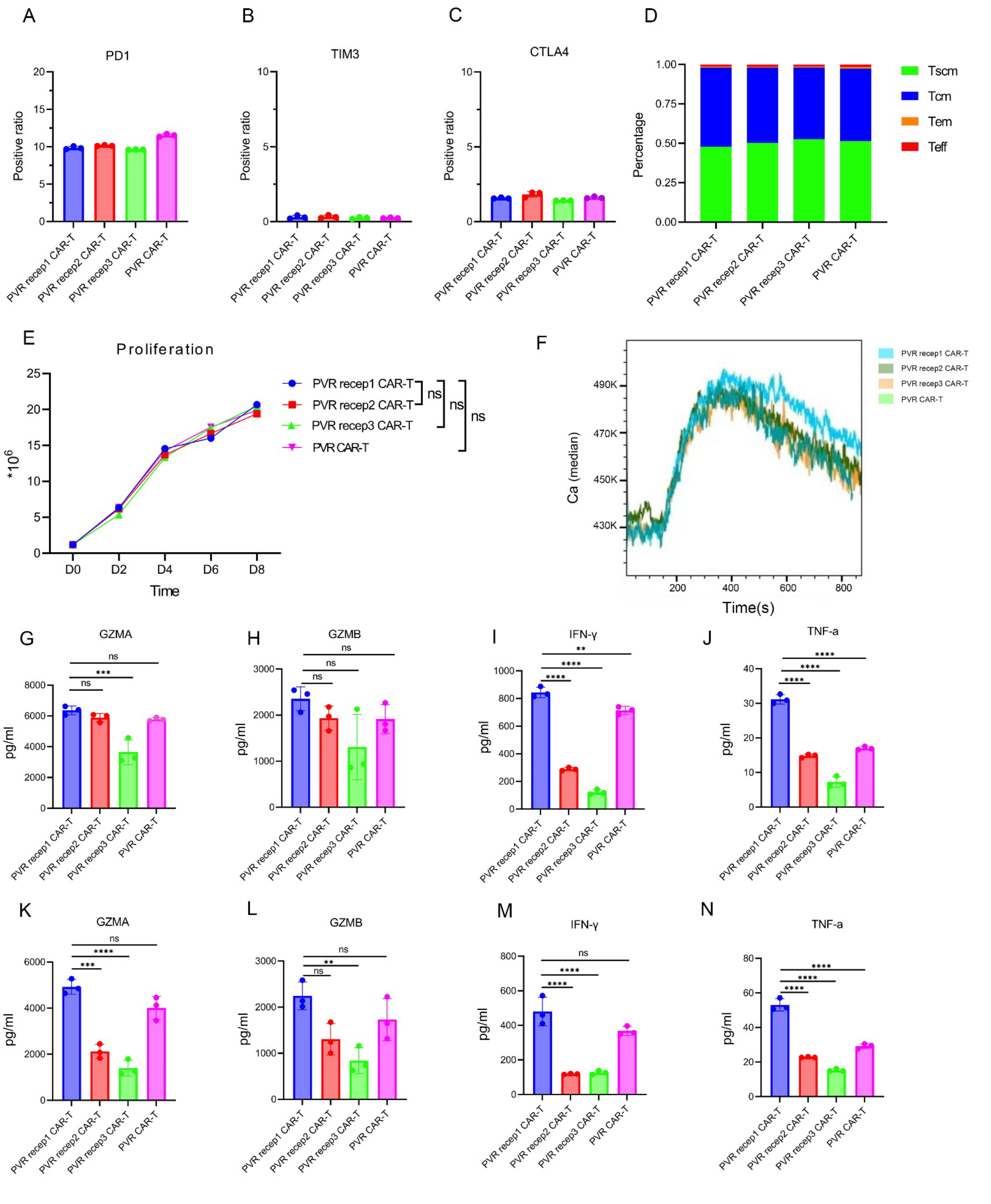

Supplementary Figure 2. The phenotype of PVR targeting CAR-T cells and efficacy against GSCs *in vitro*. A-C) Profiles of the exhaustion markers including PD1(**A**), TIM3(**B**) and CTLA4(**C**) of CAR-T cells at four days after lentiviral transduction. **D**) Phenotypic analysis of CAR-T cells showing the frequencies of stem cell memory, central memory, effector memory and effector T cells, as defined by CD45RA and CD62L surface expression. **E**) Proliferation curve of CAR-T cells after

lentiviral transduction without antigen stimulation. **F**) Analysis of intracellular calcium mobilization after stimulation with recombinant human PVR protein at 1 μg/mL. Data is representative of two independent experiments. **G-J**) Secretion of GZMA (**G**), GZMB (**H**), IFN-γ (**I**), TNF-α (**J**) of CAR-T cells after 24 h co-culturing with BNI-19-1-S. **K-N**) Secretion of GZMA (**K**), GZMB (**L**), IFN-γ(**M**), TNF-α (**N**) of CAR-T cells after 24 h co-culturing with BNI-21-1-S.

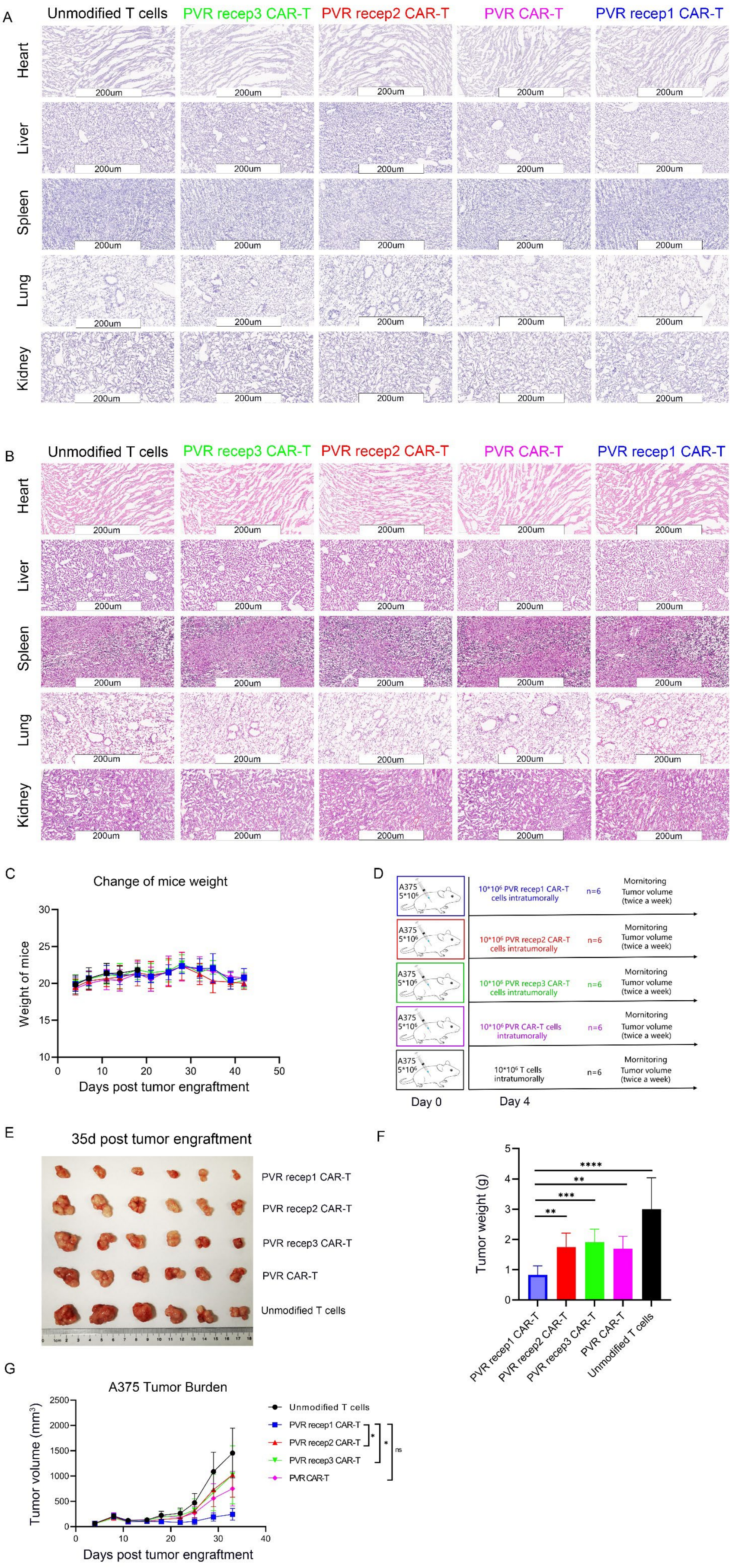
A
Unmodified T cells
PVR recep3 CAR-T
PVR recep2 CAR-T
PVR CAR-T
PVR recep1 CAR-T
Heart
Liver
Spleen
Lung
Kidney
200um
B
Unmodified T cells
PVR recep3 CAR-T
PVR recep2 CAR-T
PVR CAR-T
PVR recep1 CAR-T
Heart
Liver
Spleen
Lung
Kidney
200um
C
Change of mice weight
Weight of mice
Days post tumor engraftment
D
A375 5*10^6
10*10^6 PVR recep1 CAR-T cells intratumorally
10*10^6 PVR recep2 CAR-T cells intratumorally
10*10^6 PVR recep3 CAR-T cells intratumorally
10*10^6 PVR CAR-T cells intratumorally
10*10^6 T cells intratumorally
n=6
Mornitoring Tumor volume (twice a week)
Day 0
Day 4
E
35d post tumor engraftment
PVR recep1 CAR-T
PVR recep2 CAR-T
PVR recep3 CAR-T
PVR CAR-T
Unmodified T cells
F
Tumor weight (g)
****
**
***
**
PVR recep1 CAR-T
PVR recep2 CAR-T
PVR recep3 CAR-T
PVR CAR-T
Unmodified T cells
G
A375 Tumor Burden
Tumor volume (mm³)
Days post tumor engraftment
Unmodified T cells
PVR recep1 CAR-T
PVR recep2 CAR-T
PVR recep3 CAR-T
PVR CAR-T
*
*
ns

Supplementary Figure 3. The safety of PVR targeting CAR-T cells and *in vivo* antitumor activity. A) Immunohistochemical evaluation of CAR-T infiltration via anti-FLAG staining in organ specimens from mice treated with CAR-T cells at week 6 or unmodified T cells at day19. scale bar: 200 μm. **B)** Hematoxylin-eosin staining of organ specimens from mice treated with CAR-T cells at week 6 or unmodified T cells at day19. scale bar: 200 μm. **C)** Bodyweight changes of mice after CAR-T cells infusion. **D)** Schematic of melanoma subcutaneous models using A375 cells. **E, F)** Images of tumor **(E)** and tumor weights **(F)** of mice at 35 d after tumor engraftment. (n = 6 per group). **G)** Tumor volumes were assessed twice a week after tumor engraftment.

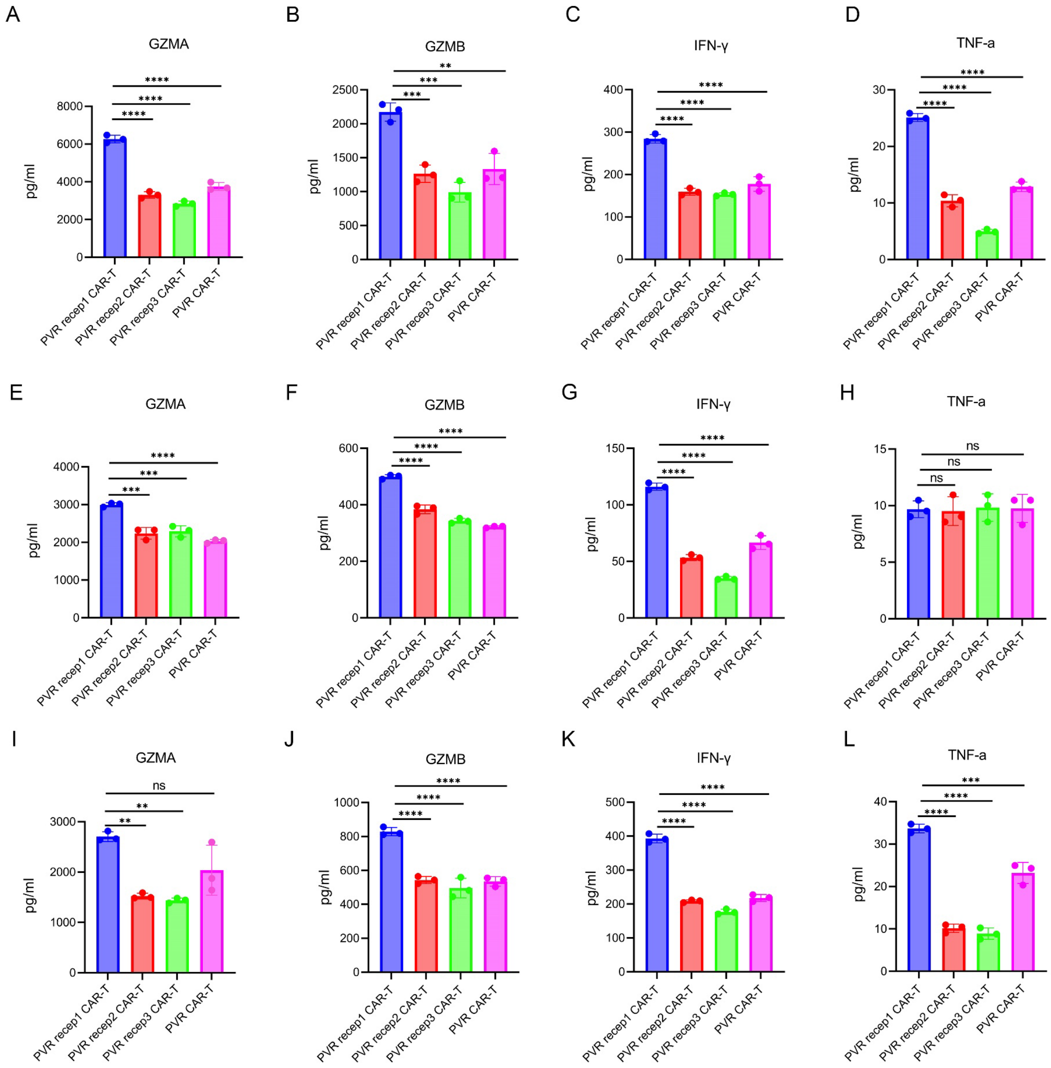

Supplementary Figure 4. Cytokines secretion of CAR-T cells co-culturing with GSCs. A-D) Secretion of GZMA (**A**), GZMB (**B**), IFN-γ (**C**), TNF-α (**D**) of CAR-T cells after 24 h co-culturing with PVR^{mod} BNI-19-1-S. **E-H**) Secretion of GZMA (**E**), GZMB (**F**), IFN-γ(**G**), TNF-α (**H**) of CAR-T cells after 24 h co-culturing with PVR^{lo} BNI-19-1-S. **I-L**) Secretion of GZMA (**I**), GZMB (**J**), IFN-γ(**K**), TNF-α (**L**) of CAR-T cells after 24 h co-culturing with PVR^{lo} BNI-21-1-S.

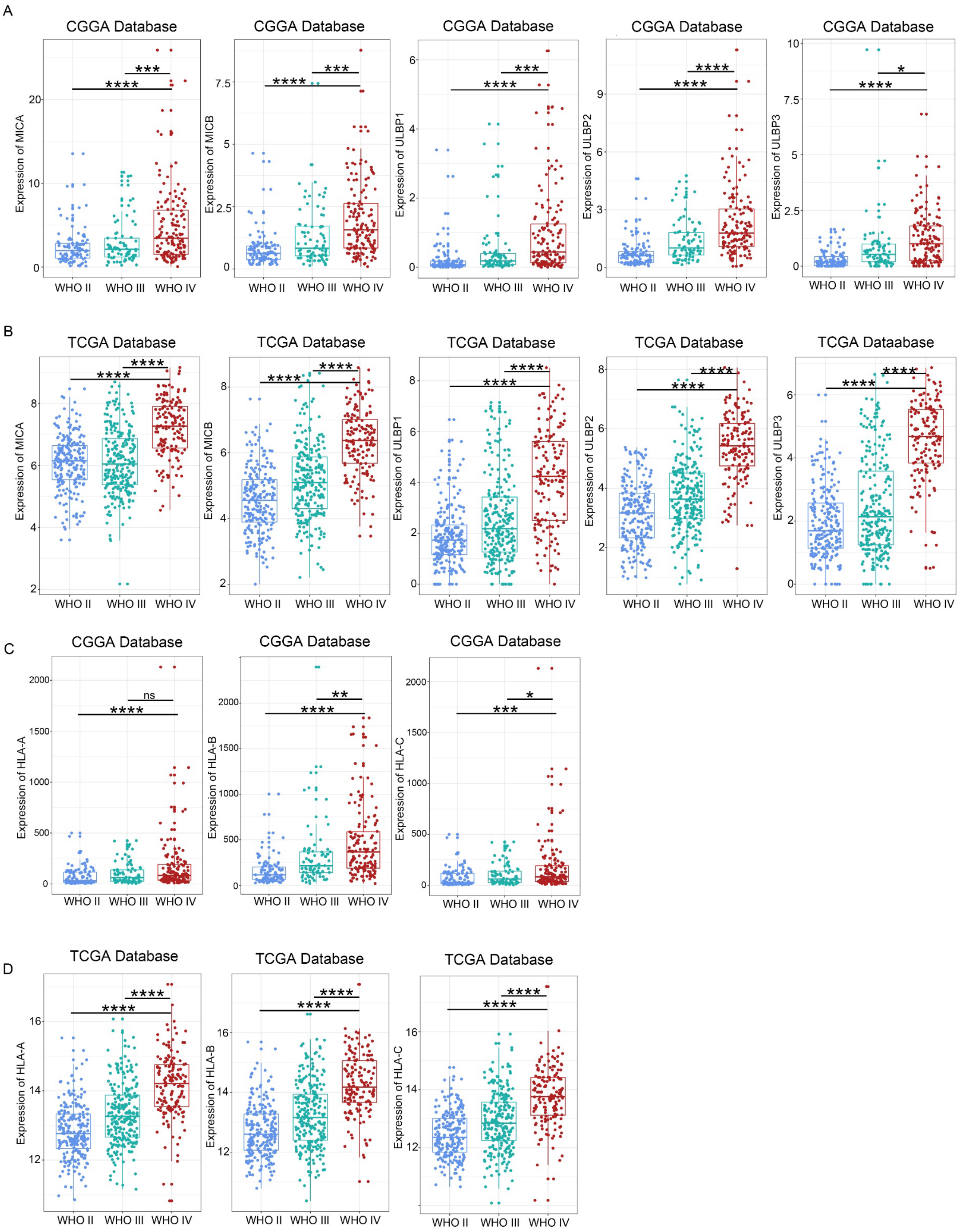

Supplementary Figure 5. Bioinformatic analysis of NKG2D ligands and MHC I molecules expression. A, B) Transcriptome sequencing data of GSCs unveiled the expression of the NKG2D ligands including MICA, MICB and ULBPs in the CCGA (**A**) and TCGA (**B**) databases. **C, D)** Transcriptome sequencing data of GSCs unveiled the expression of MHC I molecules in the CCGA (**C**) and TCGA (**D**) databases.

Supplementary Video 1. Live cell imaging of co-cultured PVR recep1 CAR-T cells and GSCs over the course of 48 h. red stained cells: GSCs; green stained cells: PVR recep1 CAR-T cells.

Supplementary
Video 1.mp4
